# *Helicobacter pylori* infection in humans and phytotherapy, probiotics, and emerging therapeutic interventions: a review

**DOI:** 10.3389/fmicb.2023.1330029

**Published:** 2024-01-10

**Authors:** Mengkai Liu, Hui Gao, Jinlai Miao, Ziyan Zhang, Lili Zheng, Fei Li, Sen Zhou, Zhiran Zhang, Shengxin Li, He Liu, Jie Sun

**Affiliations:** ^1^College of Life Sciences, Qingdao University, Qingdao, China; ^2^First Institute of Oceanography Ministry of Natural Resources, Qingdao, China; ^3^National Engineering Research Centre for Intelligent Electrical Vehicle Power System (Qingdao), College of Mechanical and Electronic Engineering, Qingdao University, Qingdao, China

**Keywords:** *Helicobacter pylori*, infection population, transmission routes, infection mechanism, phytotherapy, probiotics, emerging technologies, therapeutic mechanism

## Abstract

The global prevalence of *Helicobacter pylori (H. pylori)* infection remains high, indicating a persistent presence of this pathogenic bacterium capable of infecting humans. This review summarizes the population demographics, transmission routes, as well as conventional and novel therapeutic approaches for *H. pylori* infection. The prevalence of *H. pylori* infection exceeds 30% in numerous countries worldwide and can be transmitted through interpersonal and zoonotic routes. Cytotoxin-related gene A (CagA) and vacuolar cytotoxin A (VacA) are the main virulence factors of *H. pylori*, contributing to its steep global infection rate. Preventative measures should be taken from people’s living habits and dietary factors to reduce *H. pylori* infection. Phytotherapy, probiotics therapies and some emerging therapies have emerged as alternative treatments for *H. pylori* infection, addressing the issue of elevated antibiotic resistance rates. Plant extracts primarily target urease activity and adhesion activity to treat *H. pylori*, while probiotics prevent *H. pylori* infection through both immune and non-immune pathways. In the future, the primary research focus will be on combining multiple treatment methods to effectively eradicate *H. pylori* infection.

## 1 Introduction

*Helicobacter pylori* is a micro anaerobic, Gram-negative, spiral shape bacterium, which requires rigorous growth conditions (Flores-Treviño et al., 2018; Hirukawa et al., 2018). In 1983, *H. pylori* was first time successfully isolated from gastric mucosa biopsies of patients who had chronic antral gastritis (Isaacson and Wright, 1983). It is closely related to gastrointestinal diseases such as gastritis, gastric ulcer, and gastric cancer (Sharndama and Mba, 2022). In 2017, the World Health Organization’s International Agency for Research on Cancer published a preliminary list of carcinogens, and *H. pylori* (infection) was classified as a Class I carcinogen.

The global *H. pylori* infection rate is extremely steep. The infection rate of *H. pylori* in developing countries is 85%–95%, which is significantly higher than that in developed countries (30%–50%). Similarly, the *H. pylori* infection rates in economically underdeveloped areas are higher than those in financially developed areas. This discrepancy may be attributed to various factors such as health conditions, socioeconomic status, race, and population density (Khoder et al., 2019). Chronic smoking, inadequate vitamin supplementation, excessive daily salt intake, and host factors can all alter the acidic environment in the stomach and increase susceptibility to *H. pylori* infection among this group of people (Kayali et al., 2018).

The traditional treatment for *H. pylori* infection is proton pump inhibitors (PPI) combined with two antibiotics and bismuth (Savoldi et al., 2018). However, the rate of antibiotic resistance has increased in recent years, which led to a decline in *H. pylori* eradication rates. According to the World Health Organization (WHO), antibiotic resistance rates such as clarithromycin and metronidazole have reached unacceptable levels (more than 15%; Savoldi et al., 2018). The rate of resistance to clarithromycin was 15.6% in the early 2000s, but increased to 40.0% by the 2020s. The resistance rate of metronidazole also showed an increasing trend, from 57.8% in the early 21st century to 77.5% in the 2020s (Garvey et al., 2023). However, the eradication rate of *H. pylori* with traditional triple therapy is already below 80% (Savoldi et al., 2018). The Maastricht IV/ Florence Consensus Report states that triple therapy containing antibiotics should be abandoned when antibiotic resistance rates are higher than 15%. Therefore, quadruple therapy containing bismuth salts (two antibiotics, PPI, bismuth salts) can be used for first-line treatment (Alkim et al., 2017). But quadruple therapy still contains antibiotics and is not suitable for antibiotic-resistant people. Bismuth salts are also limited, so quadruple therapy containing bismuth is still not available as first-line therapy in countries where bismuth use is restricted (Goderska et al., 2018). Additionally, antibiotics may induce adverse effects on the human gastrointestinal tract, such as diarrhea, anorexia, emesis, abdominal distension and pain (Poonyam et al., 2019). Antimicrobial therapy is not recommended for the elderly, children, pregnant women and lactating women for safety reasons. As a result, there is a growing demand for alternative treatments that can effectively manage *H. pylori* infection. Compared with antibiotic therapy, phytotherapy and probiotics therapies produce fewer resistant strains and can reduce the side effects of antibiotics. In addition, phytotherapy and probiotics therapies are useful dietary treatments. Mozaffarian et al. point out that food is medicine (Mozaffarian et al., 2022). Foods related to plant extracts and probiotics are on the rise. These novel approaches are effective in treating *H. pylori* infection.

This paper provides a comprehensive review of the global prevalence, transmission routes, pathogenesis of *H. pylori* infection, as well as an overview of phytotherapy and probiotics therapies and emerging therapies, including their possible mechanisms against *H. pylori*. And aims to enhance the understanding of the transmission pathways and impact on human health caused by *H. pylori* infection, as well as to provide novel insights into treatment approaches and strategies for managing this pathogen.

## 2 Study on *Helicobacter pylori* infection

### 2.1 Population of *Helicobacter pylori* infection

**Figure 1:**
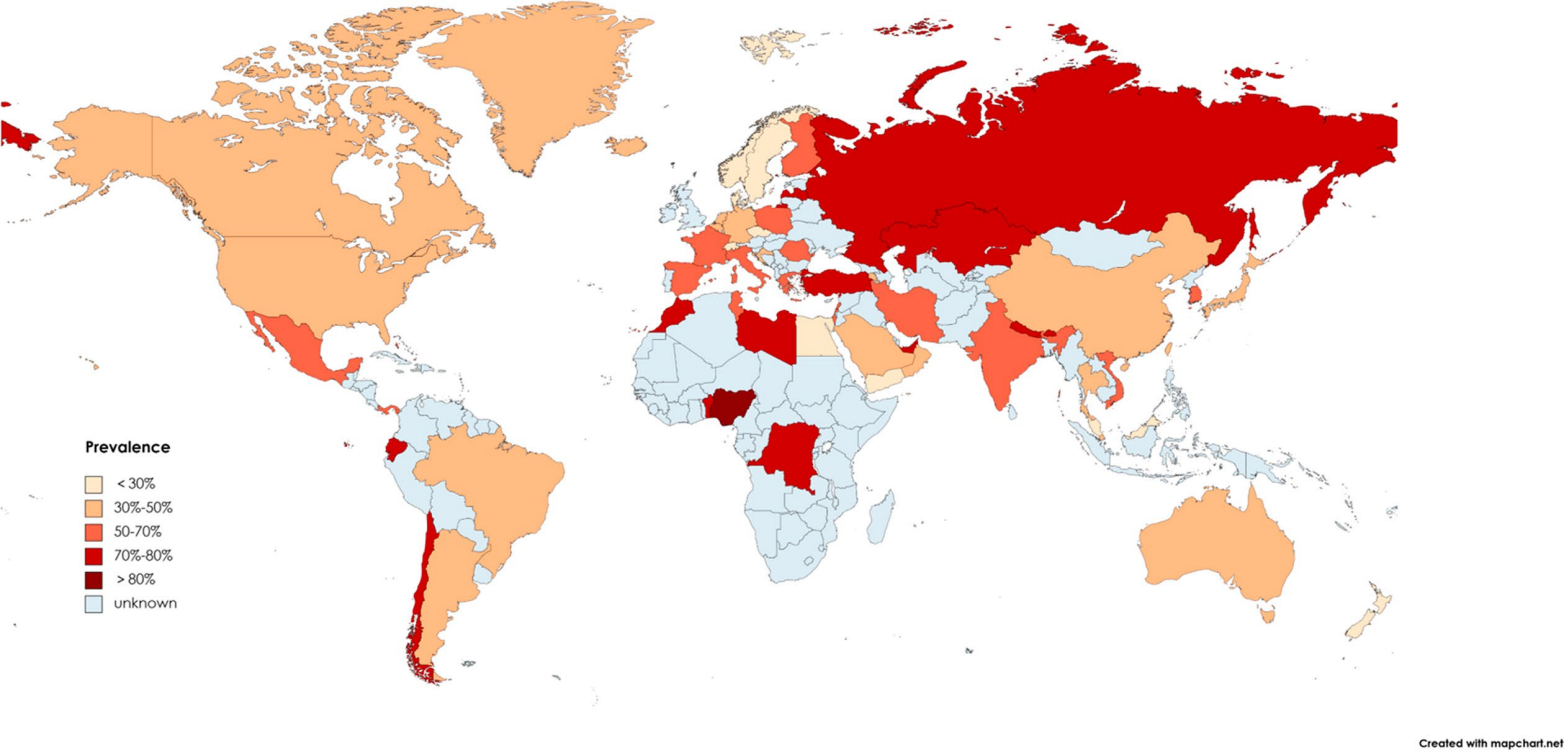
Map of the prevalence of *Helicobacter pylori* in some countries from 1970 to 2022.

*Helicobacter pylori* infection exhibits high prevalence rates in many countries, affecting nearly one-third of adults worldwide (Bruno et al., 2018). There are some geographical differences in *H. pylori* infection. Several report data related to the incidence of *H. pylori* worldwide show that the infection rate of *H. pylori* varies greatly among continents due to their economic development, health conditions, education level and eating habits, and the results are shown in Figure 1 (Rehnberg-Laiho et al., 2001; Bakka and Salih, 2002; Bener et al., 2006; Romero et al., 2007; Mansour et al., 2010; Adlekha et al., 2013; Alvarado-Esquivel, 2013; Bastos et al., 2013; Benajah et al., 2013; Hanafi and Mohamed, 2013; Krashias et al., 2013; Lim et al., 2013; Mana et al., 2013; Olokoba et al., 2013; Ozaydin et al., 2013; Pacheco et al., 2013; Sethi et al., 2013; Sodhi et al., 2013; Vilaichone et al., 2013; Eshraghian, 2014; Hooi et al., 2017; Zamani et al., 2018; Mezmale et al., 2020; Varga et al., 2020; Mežmale et al., 2021; Mentzer et al., 2022; Ren et al., 2022). According to available data, *H. pylori* prevalence is highest in Africa, followed by Asia and Europe, and lowest in the Americas and Oceania. In Africa, the infection rate of *H. pylori* is even as high as 90% in Libya, Egypt, Nigeria and other countries (Sathianarayanan et al., 2022). In China, a systematic analysis of *H. pylori* infection rate from 1990 to 2019 showed that the prevalence of *H. pylori* was close to 45%, and it was estimated that nearly 600 million people in China were infected with *H. pylori* (Ren et al., 2022). The prevalence rates of *H. pylori* in the northwest and east of China were the highest, accounting for 51.8 and 47.7%, respectively (Ren et al., 2022). In the United States, there are variations in *H. pylori* infection rates among different races and ethnicities. Notably, Hispanics exhibit the highest prevalence of *H. pylori* infection at 60.2% within the country. More than half of blacks are infected, while the prevalence rate of whites is only 21.9% (Peng et al., 2019). This phenomenon can potentially be attributed to factors such as local dietary patterns, living environments, and economic development.

The prevalence of *H. pylori* in various countries is decreasing over time as people’s living standards and eating habits have improved (Hooi et al., 2017). The prevalence of *H. pylori* in China dropped obviously from 58.3% in 1983–1994 to 40.0% in 2015–2019 (Ren et al., 2022). In Japan, the prevalence of older people born before the 1950s was more than 80%, and by 1990 the prevalence had decreased to less than 10%. Children born after the 21st century have an even lower prevalence (less than 2%; Inoue, 2017).

Numerous studies have shown that *H. pylori* infection is greatly related to age. *H. pylori* infection rates are very high in people over 60 (95%), whereas the infection rates is low in adolescents (35%; Shi et al., 2018). In Armenia, the prevalence of *H. pylori* varies with age: 18–25 years old: 13.6%; 26–45 years old: 37.9%; 46–65 years old: 61.4%; over 65 years old: 83.3% (Gemilyan et al., 2019). Therefore, we need to strengthen the research on *H. pylori* infection to totally eradicate *H. pylori* infection and prevent *H. pylori* recurrence.

### 2.2 Routes of *Helicobacter pylori* infection

To enhance the protection of humans against *H. pylori* infection, it is imperative to comprehend its routes of transmission more comprehensively. *H. pylori* infects humans through three routes:

#### 2.2.1 Person-to-person routes

The transmission of *H. pylori* can occur through person-to-person routes, especially within families where maternal-child transmission is prevalent (Yang et al., 2023). Studies have shown that *H. pylori* infection can occur in clusters in families. According to clinical investigations, if parents are infected with *H. pylori*, their children will also be infected (Ding et al., 2022). In the medical industry, the infection rate of *Helicobacter pylori* among digestive tract endoscopists is 82.4%. The infection rate of digestive tract nurses is 16.8%, while that of dentists is as high as 70% (Kheyre et al., 2018). In summary, occupational factors are also an essential pathway for possible infection with *H. pylori*.

#### 2.2.2 Animal-to-human routes

Animal-to-human transmission is thought to be an important route by which *H. pylori* infects humans. *H. pylori* is a pathogen that can infect both humans and animals (Duan et al., 2023). In the Tatra Mountains of Poland, the prevalence of *H. pylori* was particularly high among shepherds and their family members (97.6% and 86%, respectively), compared with 65.1% among farmers who were not exposed to sheep (Papież et al., 2003; Soloski et al., 2022). In addition, *H. pylori* has been detected in milk, meat (mutton, beef) and other fresh foods, suggesting that milk and sheep milk may be a vector for *H. pylori* infection in humans (Hemmatinezhad et al., 2016; Shaaban et al., 2023). A study in Japan confirmed by PCR (Polymerase chain reaction) that two dogs were infected with the same strain of *H. pylori* as their owners (Kubota-Aizawa et al., 2021).

#### 2.2.3 Food and water routes

Finally, it has been shown that *H. pylori* can be contracted through water and food infection. Fecal matter containing *H. pylori* can contaminate lakes, rivers and groundwater, which are important sources of drinking water, so it is likely that people contracted *H. pylori* through drinking water (Duan et al., 2023). Elevated levels of *H. pylori* detected in bottled water in Iran (up to 50%; Ranjbar et al., 2016). More recently, Monno et al. suggested through a meta-analysis that *H. pylori* infection was associated with dependence on external municipal water (Monno et al., 2019). There may be a risk of *H. pylori* infection from drinking externally contaminated water sources. Therefore, strengthening water source testing and enhancing dietary supervision can effectively interrupt the transmission of *H. pylori*.

If water contaminated with *H. pylori* is used to irrigate farmland, it will pose a significant risk of infecting fruits and vegetables, leading to food-borne infections in humans. Hemmatinezhad et al. detected 50 fruit salads by PCR, and found that *H. pylori* was detected in 14 samples. This may be related to direct contact with water sources, and thorough washing can mitigate the risk of infection (Hemmatinezhad et al., 2016). The 600 raw meat samples were randomly taken from slaughterhouses in different regions of Iran for detection of *H. pylori*. The contamination rate of mutton was as steep as 13.07%, and that of goat mutton was 11.53% (Mashak et al., 2020). Shaaban et al. detected *H. pylori* in 13 milk samples from farm animals infected with *H. pylori*, and *H. pylori* in 5 milk samples (Shaaban et al., 2023).

**Figure 2:**
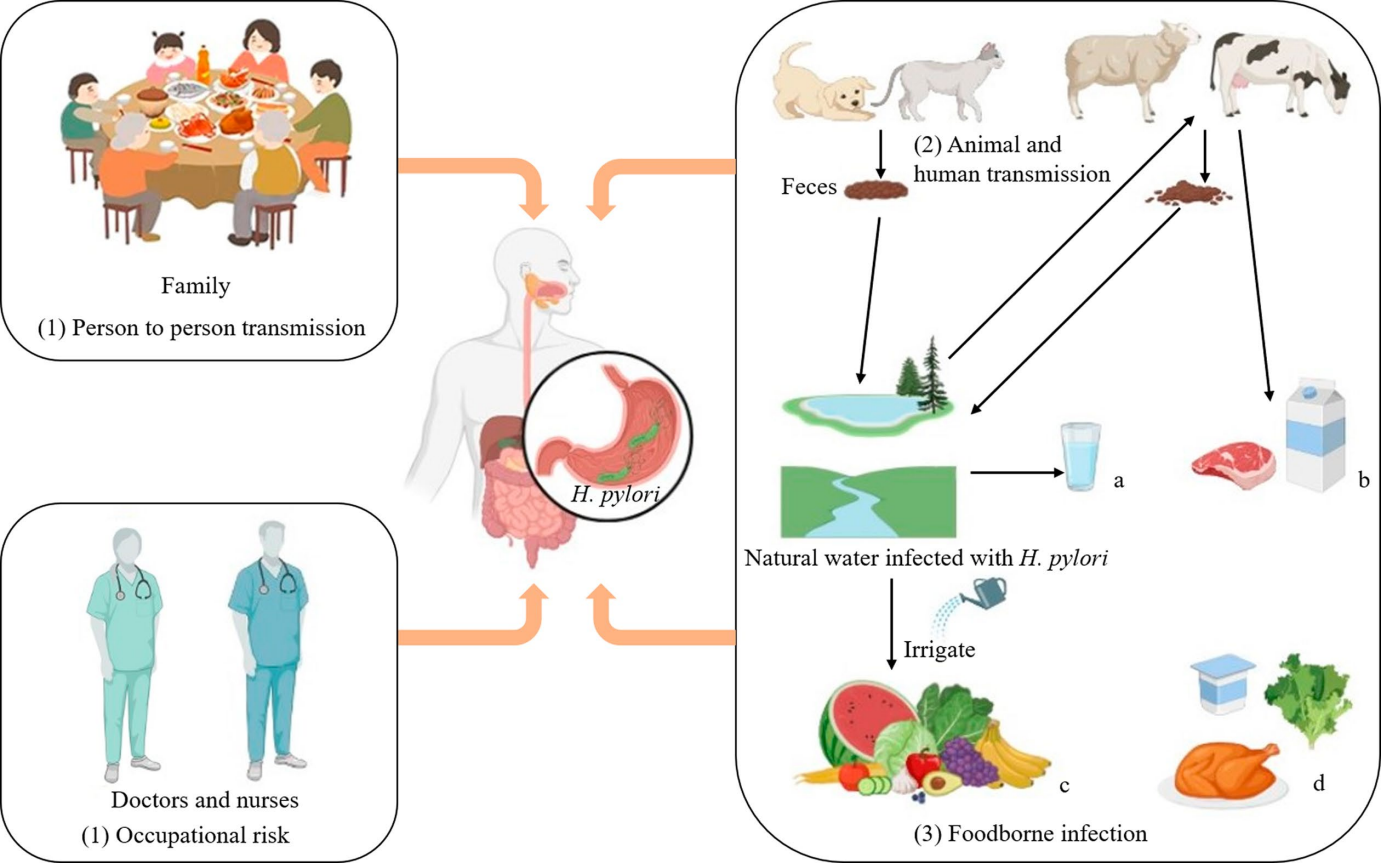
Transmission routes of *Helicobacter pylori*. Transmission routes of *Helicobacter pylori*. (1) Person to person transmission: It often spreads in families and is most likely to infect the elderly and adolescents. Occupational risk: Close contact between doctors and nurses and patients infected with *Helicobacter pylorii* may increase the odds of contracting the disease. (2) Animal and human transmission: Homeowners can contract *Helicobacter pylori* through close contact with their pets. (3) Foodborne infection: a: Fecal matter is a significant cause of contamination in most drinking water sources. b: Cattle and sheep can drink water infected with *Helicobacter pylori*, and their feces can in turn contaminate lakes and rivers. d: *Helicobacter pylori* can exist in low-acid, low-temperature, elevated humidity environments, such as chicken, raw vegetables, yogurt, and other ready-to-eat foods. Thus *Helicobacter pylori* can infect human through food and water.

Overall, as shown as depicted in Figure 2, *H. pylori* infects humans through three primary routes. Human infection with *H. pylori* can occur via water and food sources, while contact with infected people and animals escalates the risk of transmission. Therefore, it is crucial to prioritize personal hygiene in our daily lives through washing our hands frequently and disinfecting frequently, as these measures can effectively mitigate the risk of *H. pylori* transmission. Consequently, rigorous water testing is conducted in daily life to eradicate the source of *H. pylori* infection. It is essential to thoroughly cleanse vegetables and fruits prior to consumption while minimizing the consumption of raw produce and meat products.

### 2.3 Mechanism of *Helicobacter pylori* infection

**Figure 3:**
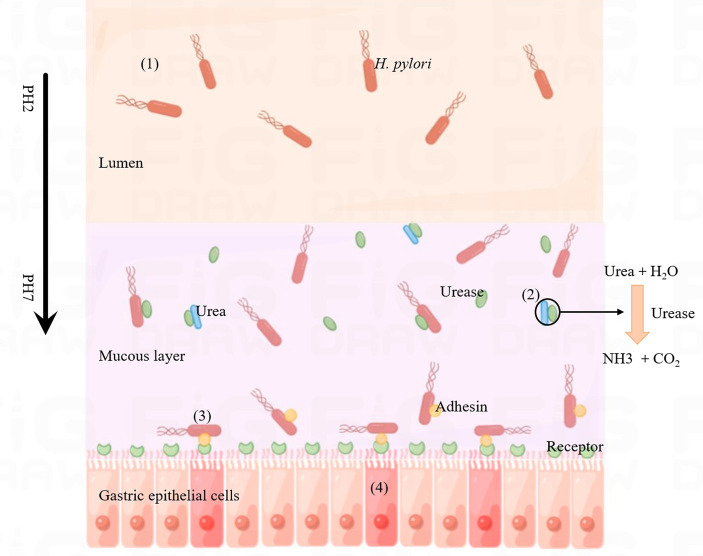
Process of *Helicobacter pylori* infection. (1) *Helicobacter pylori* enters the lumen of the host stomach. (2) *Helicobacter pylori* releases urease, decomposes the metabolites of other microorganisms (urea) into ammonia, changes the acidic environment of the stomach, which is conducive to the growth of *H. pylori*. (3) *Helicobacter pylori* releases adhesins (such as BabA, SabA, AlpA, HopQ, HopZ and OipA) that bind to specific receptors on gastric epithelial cells. (4) *Helicobacter pylori* releases CagA and VacA, which invade gastric epithelial cells and cause inflammatory reactions.

*Helicobacter pylori* infection can cause a variety of gastrointestinal diseases, which may be related to its distinct infection and colonization mechanisms. The pathogenic process is illustrated in Figure 3. After *H. pylori* invades the human stomach, it first releases urease to break down urea in the stomach to produce ammonia, which raises the pH of the stomach and provides a suitable environment for *H. pylori* to grow. The second step is the movement of *H. pylori* onto human gastric epithelial cells via flagella rotation. In the third step, *H. pylori* releases bacterial adhesins that can bind to specific receptors in gastric epithelial cells and colonize the host. Finally, *H. pylori* releases toxins, including CagA and VacA, which ultimately lead to inflammation in the gastric epithelial cells, leading to disease (Kao et al., 2016). The virulence factors CagA and VacA play a key role in the above steps.

#### 2.3.1 CagA

CagA is a protein encoded by Cag pathogenic island (Cag PAI) in *H. pylori*, with a total length of about 120–145 KDa. Cag PAI is a 40 kb locus containing a variety of genes, which can encode a type IV secretory system (T4SS), in which *H. pylori* contains a variety of adhesion hormones, including BabA (B), SabA, AlpA (B), HopQ, HopZ, and OipA, etc. They can mediate *H. pylori* to adhere tightly to gastric epithelial cells and promote the formation of T4SS (Takahashi-Kanemitsu et al., 2020). T4SS can deliver CagA to the gastric epithelial cells of the host through receptors for colonization, which cause diseases (Ray et al., 2021). Translocated CagA is localized to the interior of the plasma membrane of gastric epithelial cells, followed by phosphorylation on gastric epithelial cells. If injected into the cytoplasm via T4SS, CagA can alter host cell signaling in both phosphorylation-dependent and phosphorylation-independent ways. Phosphorylated CagA binds to phosphatase SHP-2 and affects cell adhesion, diffusion, and migration (Kao et al., 2016). Almost 60% of *H. pylori* strains detected in some Western countries and regions are Cag PAI positive (Nejati et al., 2018). The prevalence of non-cardiac gastric adenocarcinoma (AGS) cells is also elevated in Alaska. *H. pylori* infection is an essential factor in gastric adenocarcinoma. Among indigenous peoples with elevated rates of stomach cancer, Miernyk et al. found that more than half of *H. pylori* had whole CagPAI (Miernyk et al., 2020).

#### 2.3.2 VacA

Nearly all strains of *H. pylori* contain the VacA gene, which encodes the VacA protein. In mouse models, VacA has been reported to play an influential role in the initial colonization of the host (Chauhan et al., 2019). VacA is a key cytotoxin of *H. pylori* with the ability to induce cell vacuoles (Ansari and Yamaoka, 2019). After receiving a signal, VacA cleans its N-terminal and C-terminal structure, generating an N-terminal signal sequence (33 residues), a mature 88 kDa secretory toxin (p88), a short secretory peptide structure of unknown function, and a C-terminal auto-transport domain. The mature p88 is divided into two subunits (p33 and p55). p33 plays a key role in cytoplasmic membrane insertion and p55 is needed for toxins to bind to the plasma membranes. The latter part of the VacA gene signal sequence has a variable region, according to which *H. pylori* can be divided into s1 and s2. This variable region helps to recognize the intima receptor of the target cells. There are m1 and m2 alleles in p55. Typically, *H. pylori* strain s1 can secrete additional VacA, and thus genotypes with s1 / m1 combination have elevated vacuole formation capacity (Ansari and Yamaoka, 2019). Among different *H. pylori* strains, the strain with s1/ m1 allele is more closely associated with gastric epithelial injury and gastric ulcer (Ansari and Yamaoka, 2019). A meta-analysis in central Asia found that strains of *H. pylori* with the s1 / m1 combination made individuals more susceptible to stomach cancer (Liu et al., 2016). VacA antibodies have been associated with an increase in the incidence of ulcers of the digestive system, such as gastric and duodenal ulcers. The antibody has also been linked to stomach cancer (Li et al., 2016).

*Helicobacter pylori* secretes VacA near the plasma membrane of the target cell. VacA then binds to the plasma membrane to form anion-specific channels with low conductivity (Sharndama and Mba, 2022). These channels release mediators such as anions from the cell’s cytoplasm to support bacterial growth. Secreted toxins are slowly endocytosed, causing damage to the host cell’s organelles (endoplasmic reticulum, mitochondria; McClain et al., 2017). VacA can also inhibit the activation of immune cells (T lymphocytes) and affect the normal immune response. In addition, VacA can activate the autophagy pathway and promote apoptosis in gastric gland cells (Ansari and Yamaoka, 2019; Sharndama and Mba, 2022).

## 3 Main treatment methods

### 3.1 Antibiotic therapy

**Figure 4:**
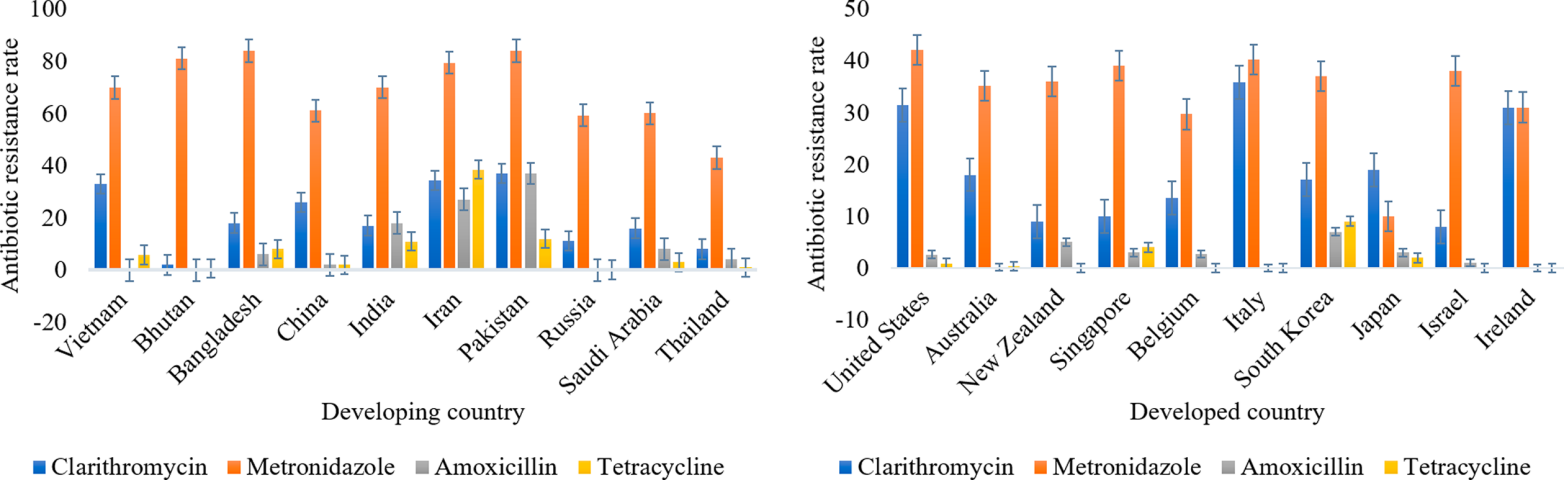
Antibiotic resistance rates in developed and developing countries.

Antibiotic therapy was the primary treatment method for *H. pylori* infection. Antibiotics commonly used to treat *H. pylori* include amoxicillin, clarithromycin, metronidazole, tetracycline, and others. The common treatment for *H. pylori* infection was a triple treatment with a PPI that reduces stomach acid production and two antibiotics (Lee et al., 2022). A meta-analysis conducted among residents of a district in Bulgaria showed that *H. pylori* strains were 42.0% resistant to metronidazole and 30% resistant to clarithromycin (Boyanova et al., 2023). Savoldi et al. conducted a meta-analysis of clarithromycin resistance rates in different regions of the world for *H. pylori*. The results showed that the resistance rate of clarithromycin in Europe was approximately 18%, and the resistance rates in Mediterranean and western Pacific were over 30% (33% and 34% respectively; Lin et al., 2023). Antibiotic resistance rates are different in developed and developing countries, as shown in Figure 4 (Boyanova et al., 2010; Binh et al., 2013; Kuo et al., 2017; Saniee et al., 2018; Savoldi et al., 2018; Maev et al., 2020; Vilaichone et al., 2020; Akar et al., 2021; Ho et al., 2022; Schubert et al., 2022; Vanden Bulcke et al., 2022). Resistance to clarithromycin and metronidazole appears to be higher than that to other antibiotics both developed and developing countries. Therefore, the use of antibiotics in the treatment of *H. pylori* infection should be controlled. Resistance of *H. pylori* to antibiotics is primarily associated with the formation of biofilms (Hou et al., 2022). There is a layer of extracellular polymeric substances (EPS) on the surface of microorganisms. Biofilm is a community of microorganisms, EPS, and alternative substrates that mainly contain sugar, protein, nucleic acids, and other substances. EPS, which is negatively charged on the surface, prevents antibiotics and other drugs from getting through the biofilm into the bacteria and makes the bacteria resistant to antibiotics and other drugs. Therefore, some bacteria (forming biofilm) are 1,000 times more resistant to antibiotics than planktonic microorganisms (Shen et al., 2020; Hou et al., 2022). Thus, if antibiotics are used to treat *H. pylori* while biofilm is being created, antibiotics will be blocked from biofilm. As a result, it is not possible for antibiotics or drugs to enter the target cell for treatment and ultimately leads to treatment failure. In addition, the biofilm blocks immune cells from attacking *H. pylori*, and thus causes antibiotic resistance (Hou et al., 2022). Furthermore, under the conditions of environmental deterioration and the existence of antibiotics, *H. pylori* will enter a state of viable but non-culturable (VBNC) to resist environmental changes and the invasion of antibiotics (Li et al., 2021). Both the biofilm and VBNC status of *H. pylori* can lead to its own resistance to antibiotic. Due to the development of drug resistance, *H. pylori* is not treated promptly, so the infection can produce a range of complications such as bleeding in the stomach, obstruction of the outlet of the digestive system, and perforation of the stomach. In addition, taking non-steroidal anti-inflammatory drugs can easily cause peptic ulcers, which can cause patients to suffer from stomach pain, loss of appetite, vomiting and abdominal swelling (Lanas and Chan, 2017). Previous studies have shown that *H. pylori* infection can cause indigestion in humans. After treatment in these patients successfully removed *H. pylori* from the body, there was a significant improvement in adverse digestive system reactions (Moayyedi et al., 2017). In addition, approximately four out of five stomach cancers caused by *H. pylori* are non-cardiac stomach cancers (Guevara and Cogdill, 2020). In previous studies, *H. pylori* positive patients were less likely to develop stomach cancer after eradication therapy (compared to placebo; Gawron et al., 2020). So finding a way to treat *H. pylori* without developing resistance could reduce the incidence of stomach cancer.

The drug resistance rate is higher when clarithromycin and metronidazole are used to treat *H. pylori*, so bismuth quadruple therapy is recommended as first-line treatment. When triple therapy fails, levofloxacin-based therapies, as well as therapies containing macrolides, may be used as an alternative treatment (Guevara and Cogdill, 2020). Bismuth quadruple therapy was used to solve the problem of antibiotic resistance, which consists of two antibiotics (tetracycline and metronidazole), bismuth and proton pump inhibitor (Harb et al., 2015). However, the quadruple therapy has some limitations. Bismuth salts are restricted in some countries because of their toxicity, and tetracycline has side effects. These reasons limit the large-scale use of quadruple therapy (Goderska et al., 2018). These antibiotics also have side effects. Examples include amoxicillin, clindamycin and tetracycline, which can cause diarrhea. Taking multiple antibiotics can also affect the normal flora in the stomach of the host, resulting in physical discomforts such as nausea, vomiting, dizziness, and rash (Salehi et al., 2018). Therefore, there is an urgent need to find non-antibiotic treatments for *H. pylori* infection.

### 3.2 Phytotherapy

Phytotherapy, also known as herbal therapy, is a method that applies the plant itself or plant extracts to medicine. Herbs can be used to treat various gastrointestinal diseases, reduce antibiotic resistance and side effects, and improve the cure rate for *H. pylori* (Li et al., 2023). Herbal products include raw or processed parts of plants, such as leaves, stems, flowers, roots and seeds. At present, numerous plant extracts have been reported to have therapeutic effects on *H. pylori* infection, such as *Acacia nilotica*, *Calophyllum brasiliesnse*, *Bridelia micrantha*, *Allium sativum*, *Pistacia lentiscus*, *Brassica oleracea*, *Glycyrrhiza glabra*, *Camellia sinensis*, *Cinnamomum cassia*, *Evodia rutaecarpa*, *Impatiens balsamina* and so on (Safavi et al., 2015; Liu et al., 2018; Sathianarayanan et al., 2022).

*Helicobacter pylori* infection can be treated by consuming certain plant products or certain fruits. These plants contain flavonoids, terpenoids, coumarins, essential oil, tannins and alkaloids, contributing to treat *H. pylori* infection (Abd El-Moaty et al., 2021; Guerra-Valle et al., 2022). Fahmy et al. found that flavonoids extracted from *Erythrina speciosa* (Fabaceae) showed strong inhibitory activity against *H. pylori*, with the lowest inhibitory concentration (MIC) of 31.25 μg/mL (Fahmy et al., 2020). Spósitoe et al. analyzed the components of the leaves of *Casearia sylvestris* Swartz and showed that the leaves contain a significant amount of terpenoids, which may be the key to its inhibition of *H. pylori*. Zardast et al. gave patients infected with *H. pylori* fresh garlic, and the levels of *H. pylori* in the gastric mucosa of the patients decreased significantly after 3 days (Zardast et al., 2016). The ethyl acetate part of the leaves of this plant has the best antibacterial activity, and the MIC is 62.5 μg/mL (Spósito et al., 2019). Ayoub et al. obtained the essential oil from the stem of *Pimenta racemosa*, which also had an excellent bactericidal activity against *H. pylori*, with a MIC of 3.9 μg/mL (Ayoub et al., 2022). In a mouse model, when green tea extract was administered to mice infected with *H. pylori* at a concentration of 2,000 ppm for 6 weeks, the prevalence was suppressed to the greatest extent. These plant extracts inhibit *H. pylori* infection mainly through a permeable membrane, anti-adhesion, urease inhibition and other ways (Shmuely et al., 2016). When MIC≤100 μg/mL, plant extracts were considered to have excellent antibacterial activity (Fahmy et al., 2020). As a result, plant foods can be used to treat *H. pylori* infection in people. They have shown great potential in eradicating *H. pylori* and preventing related gastric diseases caused by *H. pylori*.

Zojaji et al. added 500 mg vitamin C daily to 150 patients infected with *H. pylori* on the basis of antibiotic therapy (amoxicillin and metronidazole, bismuth and omeprazole). Finally, 79 patients in the vitamin C supplementation group were negative for rapid urease test (RUT), and the eradication rate of *H. pylori* was 78%. The eradication rate from antibiotic therapy alone was only 56.4 percent (Öztekin et al., 2021). In addition, Ibrahim et al. showed that the effect of *Pelargonium graveolens* oil combined with clarithromycin was better, and the fractional inhibitory concentration index was 0.38 mg/mL (the MIC of the essential oil was 15.63 mg/mL; Ibrahim et al., 2021). The above study shows that plants or fruits can be combined with antibiotics to enhance the therapeutic effect. As a result, the combination of plant extracts and antibiotics is beneficial to eradicate *H. pylori*. Therefore, plants may be one of the main forces in treating *H. pylori* infection in the future.

#### 3.2.1 Therapeutic mechanism of phytotherapy

The antibacterial mechanisms of phytotherapy include inhibition of urease activity, anti-adhesion activity, DNA damage, inhibition of protein synthesis and oxidative stress, which are discussed in Table 1.

**Table 1:** Effect of plant extracts on *Helicobacter pylori*.

Treatment method	Compound	Source	MIC/MBC/IZD	Ref.
Antibiotic therapy	Metronidazole	——	MIC: 2.24 μg/mL	Hassan et al. (2016)
	Amoxicillin	——	MIC: 13.50 μg/mL	Hassan et al. (2016)
			IZD: 15.0 mm	Zhang and Yue (2017)
	Tetracycline	——	MIC: 0.25 μg/mL	Feng et al. (2023)
	Clarithromycin	——	MIC: 1.95 μg/mL	Ayoub et al. (2022)
			IZD: 12.98 mm	Fagni Njoya et al. (2022)
Phytotherapy	The methanol extracts	*Alstonia boonei*	IZD: 7–36 mm	Fagni Njoya et al. (2022)
	Diterpenoids	*Icacina trichantha* (Icacinaceae)	MIC: 8–64 μg/mL	Xu et al. (2021)
	Naringenin (from essential oil)	*Cannabis sativa* L.	MIC_50_: 16 μg/mL	Zengin et al. (2018)
			MIC_90_: 32 μg/mL	
	Eugenol essential oil	*Syzygium aromaticum*	MIC: 23.0–51.0 μg/mL	Elbestawy et al. (2023)
			IZD: 10 ± 06–22 ± 04 mm	
	Essential oil	*Thymus serpyllum*	MIC: 2.0–4.0 μL/mL	Knezevic et al. (2018)
	Thyme	*Thymus vulgaris* L.	MIC: 15.6 mg/L	Korona-Glowniak et al. (2020)
			MBC: 15.6 mg/L	
	Ylang-Ylang	Thomson, Annonaceae	MIC: 15.6 mg/L	Korona-Glowniak et al. (2020)
			MBC: 62.5 mg/L	
	Oregano	*Origanum vulgare* L.	MIC: 31.3 mg/L	Korona-Glowniak et al. (2020)
			MBC: 31.3 mg/L	
	Lavender oil	*Lavandula angustifolia* Mill.	MIC: 62.5 mg/L	Korona-Glowniak et al. (2020)
			MBC: 125 mg/L	
	Carvacrol	*Satureja hortensis*	MIC: 0.13 mg/mL	Lesjak et al. (2016)
	Alkaloids	Rhizoma Coptidis	MIC: 25–50 μg/mL	Li et al. (2018)
			MBC: 37.5–125 μg/mL	
	Essential Oil	*Mentha* Cultivars	MIC of *H. pylori* strain ATCC 43504: 15.6–31.3 mg/L	Piasecki et al. (2023)
	Ethyl acetate	*Persea americana* Mill.	MIC: 128 μg/mL	Athaydes et al. (2022)
			MBC: 256 μg/mL	
	Essential oil	*Pimenta racemosa*	MIC: 50 μg/mL	Ayoub et al. (2022)
	Thymol	Lamiaceae and Apiaceae families	MIC: 4–128 μg/mL	Sisto et al. (2021)
			MBC:4–128 μg/mL	
	The methanol extracts	*Aframomum pruinosum* Gagnepain seeds	MIC: 128–512 μg/mL	Kouitcheu Mabeku et al. (2017b)
	The hydroethanolic extracts	*Arrabidaea chica* leaves	MIC: 12.5 mg/mL	Mafioleti et al. (2013)
	The ether extracts1-8	*Desmostachya bipinnata (L.)*	MIC: 12.5–50 mg/mL	Ibrahim et al. (2018)
	The methanol extracts	*Bryophyllum pinnatum* (Lam.) Kurz leaves	MIC: 32 μg/mL	Kouitcheu Mabeku et al. (2017a)
			MBC: 256 μg/mL	
	The methanol extracts	Fenugreek	MIC: 100 μg/mL	Hasna et al. (2023)
			MBC: 150 μg/mL	
	The methanol extracts	Cumin	MIC: 150 μg/mL	Hasna et al. (2023)
			MBC: 250 μg/mL	
	The ethanol extracts	White rose petal	MIC: 0.10 mg/mL	Park et al. (2016)
	The butanol extracts	White rose petal	MIC: 0.01 mg/mL	Park et al. (2016)
	Essential oil	*Thymus vulgaris*	MIC: 62.5 μg/mL	Chama et al. (2020)
	Essential oil	*Cinnamomum glanduliferum* (Wall) Meissn bark	MIC: 0.49 μg/mL	Taha and Eldahshan (2017)
	The acetone extracts	*Heterotheca inuloides* (Mexican arnica)	MIC: 31.25 μg/mL	Egas et al. (2018)
	The bark extracts	*Spathodea campanulata*	MIC: 0.125 mg/mL	Ngnameko et al. (2019)
	The leaves extracts	*Nicotina tabacum*	MIC: 1.0 mg/mL	Ngnameko et al. (2019)
	The leaves extracts	*Allanblackia florinbunda*	MIC: 2.0 mg/mL	Ngnameko et al. (2019)
	Irigenin	*Iris confusa*	MIC: 3.90 μg/mL	Abdel-Baki et al. (2022)
	1-hydroxybenzoisochromanquinone	Aerial parts of *Mitracarpus hirtus*	MIC: 0.0625 μg/mL	Xu et al. (2022)
	Benzo[g]isoquinoline-5,10-dione	Aerial parts of *Mitracarpus hirtus*	MIC: 0.125 μg/mL	Xu et al. (2022)
	Nimbolide	The neem tree (*Azadirachta indica* A. Juss)	MIC of *H. pylori* strain G27: 2.5 μg/mL	Wylie et al. (2022)
			MBC of *H. pylori* strain G27: 5 μg/mL	
	Nimbolide	The neem tree (*Azadirachta indica* A. Juss)	MIC of *H. pylori* strain 26,695: 1.25 μg/mL	Wylie et al. (2022)
			MBC of *H. pylori* strain 26,695: 10 μg/mL	
	Nimbolide	The neem tree (*Azadirachta indica* A. Juss)	MIC of *H. pylori* strain HPAG1: 2.5 μg/mL	Wylie et al. (2022)
			MBC of *H. pylori* strain HPAG1: 10 μg/mL	
	The aqueous extracts	*Alstonia boonei*	IZD: 7–35 mm	Fagni Njoya et al. (2022)
	The methanol extracts	*Alstonia boonei*	IZD: 7–36 mm	Fagni Njoya et al. (2022)
	The volatile oil of CAL	*Chenopodium ambrosioides* L. (CAL)	MIC: 16 mg/L	Ye et al. (2015)
	The dichloromethane extracts	*Parthenium hysterophorus*	MIC: 15.6 mg/mL	Espinosa-Rivero et al. (2015)
	The aqueous extracts	*Hibiscus sabdariffa* L. (Malvaceae; AEHS)	MIC: 9.18–16.68 mg/mL	Hassan et al. (2016)
	The hydroethanolic extracts	*Cochlospermum regium* (Bixaceae)	MIC: 100 μg/mL	Arunachalam et al. (2019)
	The methanol extracts	*Bryophyllum pinnatum*	MIC: 32 μg/mL	Kouitcheu Mabeku et al. (2017a)
			MBC: 256 μg/mL	
	Essential oil	*Casearia sylvestris* leaves	MIC: 125 μg/mL	Spósito et al. (2019)
	The leaf extracts	*Centella asiatica* leaves	MIC: 0.125–8 mg/mL	Zheng et al. (2016)
	Essential Oil	*Campomanesia lineatifolia* leaves	MIC: 6 μL/mL	Neves et al. (2022)
	Neem oil	*Azadirachta indica* seeds	MIC of *H. pylori* strain F40/499: 64 μg/mL	Cesa et al. (2019)
			MBC of *H. pylori* strain F40/499: 64 μg/mL	
	Trichanthol	*Icacina trichantha* (Icacinaceae)	MIC: 8–64 μg/mL	Dinat et al. (2023)
	The leaf oil	*Pachira aquatica* Aubl.	MIC: 20 μg/mL	Gamal El-Din et al. (2018)
	Essential oil	*Piper longum*	MIC: 1.95 μg/mL	Al-Sayed et al. (2021)
	Essential oil	White pepper	MIC: 3.90 μg/mL	Al-Sayed et al. (2021)
	Essential oil	*Piper nigrum*	MIC: 7.81 μg/mL	Al-Sayed et al. (2021)
	The methanol extracts	*Bergenia ciliata*	MIC: 12.50 mg/mL	Ali et al. (2020)
	The ethyl acetate fraction	*Hibiscus rosa-sinensis* red flower	MIC: 0.2–0.25 mg/mL	Ngan et al. (2021)
			MBC: 1.25–1.5 mg/mL	
	The aqueous extract (AE) and 75% hydroalcoholic extracts (HE)	The dried flower bud of *Syzygium aromaticum*	MIC_AE and HE_: 160–320 μg/mL	Peng et al. (2022)
	Naringenin	*Hibiscus rosa sinensis* L. flower	MBC: 1000 mg/L	Tran Trung et al. (2020)

MIC, Minimum inhibitory concentration; MBC, Minimum bactericidal concentration; IZD, Inhibition zone diameter.

##### 3.2.1.1 Inhibition of urease activity

After *H. pylori* infects the host, it can neutralize the acidic stomach environment through the action of urease to provide a suitable environment for its growth (Woo et al., 2021). Urease can hydrolyze urea to ammonia and bicarbonate, creating an environment suitable for the growth of *H. pylori* (Korona-Glowniak et al., 2020). Therefore, *H. pylori* infection can be treated by inhibiting urease activity. When the concentration of zerumbone (from *Zingiber zerumbet* Smith) was 20 μM, the urease activity decreased to 73% of the control group (without zerumbone treatment), and the higher the concentration of the plant, the higher inhibited the urease activity (Woo et al., 2021). Compared with the known urease inhibitors (50% inhibition concentration is 4.56 0.41 μg/mL), the IC_50_ value of *Zanthoxylum nitidum* on urease activity of *H. pylori* is 1.29 0.10 mg/mL, and additional research shows that the plant can reduce the urease level by interacting with sulfhydryl groups on urease (Lu et al., 2020). Zhou et al. found that the MIC of Palmatine (from *Coptis chinensis*) was 75–100 μg/mL when the pH was close to five through agar dilution experiment (Zhou et al., 2017). The literature has not reported any instances of *H. pylori*’s resistance to plants thus far (Sathianarayanan et al., 2022). Therefore, plant extracts as excellent antibacterial agents can be used to treat *H. pylori* infection.

##### 3.2.1.2 Anti-adhesion activity

**Figure 5:**
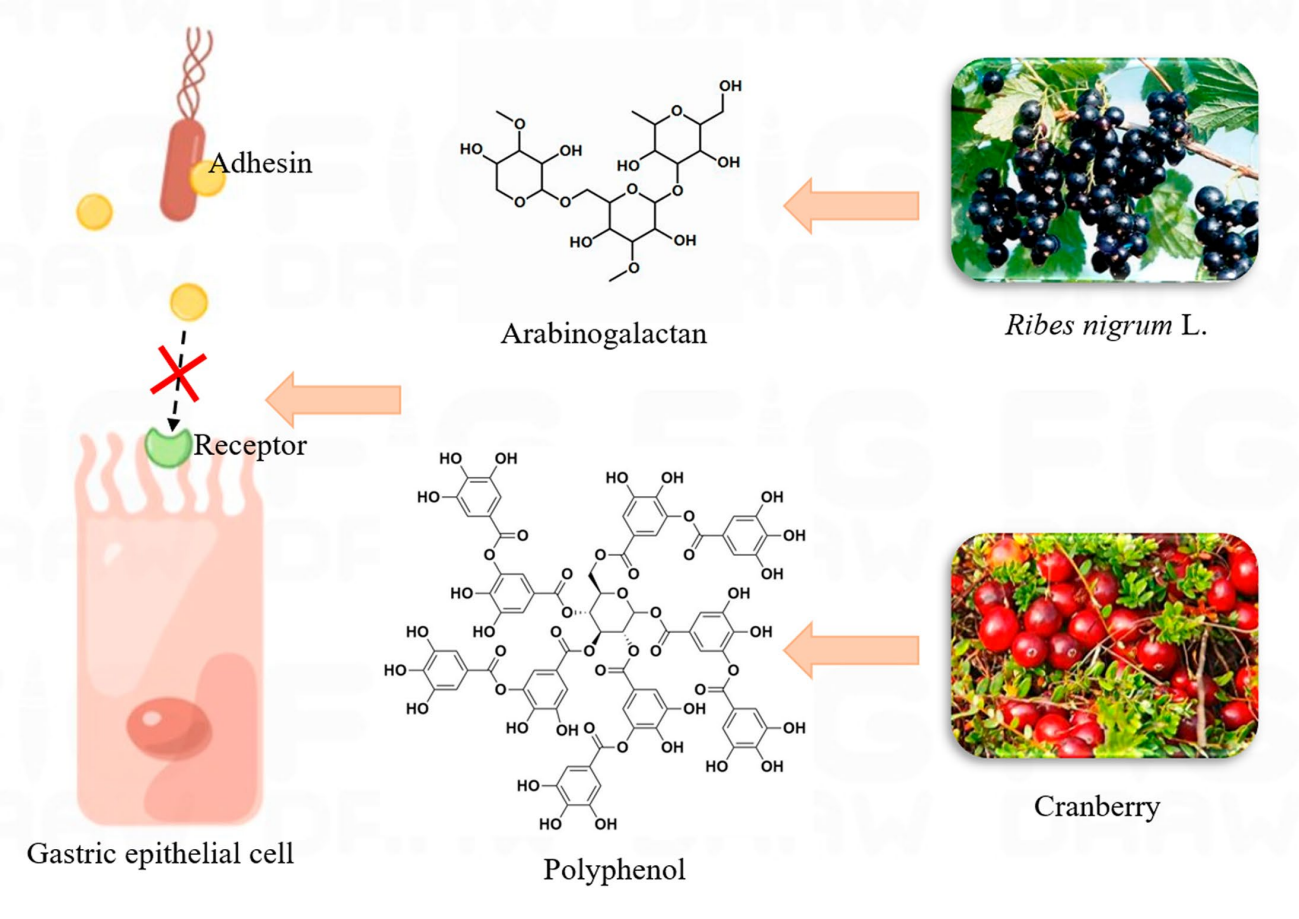
The possible mechanism of action of *Ribes nigrum* L. and cranberry.

After *H. pylori* infects the host, it first releases urease to neutralize the acidic conditions of the stomach, and then colonizes the stomach by releasing adhesin to bind to specific receptors in the stomach. Therefore, *H. pylori* infection can be treated by inhibiting *H. pylori* adhesion (Kao et al., 2016). The low molecular sulfate polysaccharides of *C. lentillifera* (CLCP-1) exhibit (at a concentration of 1,000 μg/mL) reduced *H. pylori* adherence to AGS cells by approximately 50% compared to controls not treated with the extract, with a significant reduction in cell infection rates (Le et al., 2022). Plant polysaccharides from natural products have been reported to inhibit the adhesion of *H. pylori* to gastric epithelial cells, thus preventing the formation of biofilms. Such herbs can effectively inhibit the adhesion of *H. pylori* and improve the effective treatment rate of the drug (Moghadam et al., 2021). Gottesmann et al. extracted a highly esterified saccharide from *Abelmoschus esculentus*, which could hinder the adhesion of *H. pylori* to AGS cells. The results showed that IC_50_ was 550 μg/mL (Gottesmann et al., 2020). According to the report, *Capsicum annum*, *Curcuma longa*, and *Abelmoschus esculentus* significantly impede the adhesion of *H. pylori* to AGS cells, with suppression rates exceeding 10% for all three (Yakoob et al., 2017). Wheat germ extract has been shown to treat *H. pylori* infection through its antigens. *H. pylori* can release adhesins (BabA, SabA, etc.) and bind to the target cell receptor, so as to colonize the host cell. However, the structure of this extract is similar to that of the receptor, and it competes to bind to the adhesin, which results in the failure of the adhesion factor to bind to the host cell, thus reducing *H. pylori* infection (Sun et al., 2020). Dang et al. extracted 14 therapeutic peptides from wheat germ with binding levels of −6.0 to −7.4 and −6.0 to −7.8 kcal/mol to adhesion factors released by *H. pylori*, respectively. These negative values indicate that the peptide is tightly bound to the adhesion factor (Dang et al., 2022). The above study provides a new direction for the anti-adhesion mechanism of plant-derived peptides, demonstrating that plant-derived peptides are an effective alternative for the treatment of *H. pylori*. A previous study found that oral cranberry therapy in mice already infected with *H. pylori* reduced the infection rate to 20 percent after 30 days of treatment (Xiao and Shi, 2003). Black currant (*Ribes nigrum* L.) can inhibit the adhesion of *H. pylori* through arabinogalactan, which can block the binding of adhesin to gastric epithelial cell receptors, thus affecting the invasion of *H. pylori* into the body (Lengsfeld et al., 2004; Messing et al., 2014). Figure 5 shows the possible mechanisms of action for cranberry and black currant. In host cells, plant extracts can inhibit *H. pylori* adhesion to host cells and affect the formation of the T4SS system.

##### 3.2.1.3 Oxidative stress

Recent studies have demonstrated that *H. pylori* can cause inflammation in gastric epithelial cells and ultimately induce ROS, resulting in DNA damage, which is also an influential factor in gastric cancer (Jain et al., 2021). Some plant extracts can inhibit *H. pylori* through oxidative stress. Studies have showed that 2-methoxy-1,4-naphthoquinone (MeONQ) isolated from the pods of *I. balsamina* L. had extremely strong inhibitory activity against the growth of *H. pylori*. The bacteriostatic concentration of MeONQ (0.156–0.625 μg/mL) was considerably lower than metronidazole (MIC was 160–5,120 μg/mL). When MeONQ passes through the cell membrane, it is immediately metabolized by the flavoenzymes in the cell and undergoes a series of redox reactions to produce reactive oxygen species (ROS) with strong oxidation. These ROS can additionally damage intracellular macromolecules and may indirectly lead to the death of *H. pylori*, which may be the bacteriostatic mechanism of MeONQ (Wang et al., 2011). Olive leaf extract E2 reduced ROS production by up to 33.9%, while also reducing *H. pylori* activity by about 2 log CFU/mL (Silvan et al., 2021). *H. pylori* infection induces an immune response and increases the production of pro-inflammatory cytokines IL-8 and ROS. When gastric epithelial cells containing *H. pylori* were treated with resveratrol, the synthesis of IL-8 and ROS was inhibited (Wang et al., 2020). Ayse et al. extracted a phenolic compound from celery and added it to AGS cells infected with *H. pylori* for co-culture. It was found that the compound suppressed the increase in ROS levels in a dose-dependent manner (Günes-Bayir et al., 2017). Plants can suppress the oxidative stress produced by *H. pylori*, which can reduce the damage caused to the human body by *H. pylori*.

##### 3.2.1.4 Amphiphilicity of the compound

Studies have found that drugs with both hydrophilic and hydrophobic properties can inhibit the growth of *H. pylori*, and quinolone alkaloids are thought to have this advantage. The quinolone alkaloid is an amphiphilic monocyclic monoterpenoid derived from Evodia rutaecarpa. The MIC against *H. pylori* is less than 0.05 μg/mL, which has the same inhibitory effect as commonly used antibiotics. This derivative is strongly hydrophilic and hydrophobic. Due to its hydrophilicity, the compound diffuses to the cell wall of *H. pylori* through the surrounding water, while the hydrophobicity makes the compound partially bound to the plasma membrane of the cell, resulting in the loss of membrane integrity and thus affecting the growth of *H. pylori* (Hamasaki et al., 2000).

Harmati et al. constructed a mouse model infected with *H. pylori* SS1 and treated the mice with extracts from two plants (*Satureja Hortensis* and *Origanum Vulgaris* subsp). Through PCR analysis and Giemsa staining observations, it was found that only 30% of the mice were still infected with *H. pylori* and the rest tested negative for *H. pylori* (Harmati et al., 2017). Plant extracts are a valuable resource for the treatment of *H. pylori*. Some plant extracts can be made into health foods that have been shown to have a beneficial effect on the treatment of gastrointestinal discomfort. Compared with antibiotics and PPI treatment, most of the effective ingredients in plant therapy come from plants, fruits and spices (Liu et al., 2018), which may be relatively economical in areas with poor sanitary conditions and have strong bactericidal activity and anti-inflammatory activity. Whether it is treated alone or combined with antibiotics, the effect is safe and reliable (Shmuely et al., 2016). Various studies have demonstrated the efficacy of plant extracts in the treatment of *H. pylori*, thus indicating the potential incorporation of certain botanical products into conventional therapies for *H. pylori* infection.

### 3.3 Probiotics

In microbial therapy, probiotics are commonly used to treat *H. pylori* infection. Probiotics are a kind of active microorganisms that are beneficial to the host by colonizing the human body and changing the composition of the flora in a certain part of the host. Probiotics, especially *Lactobacillus*, can be used to treat *H. pylori* infection in the stomach because they grow in acidic conditions at pH 4–6. Probiotics have safety, immunomodulatory and antibacterial advantages, so they are frequently administered alone or in combination with drugs to treat certain gastrointestinal disorders (Yang and Yang, 2019a,b). They can regulate host immune function or maintain intestinal health by regulating the balance of intestinal flora. In a randomized controlled trial, a probiotic (*Lactobacillus Acidophilus* LA-5, *Lactiplantibacillus plantarum*, *Bifidobacterium lactis* BB-12, and *Saccharomyces boulardii*) combined with four antibiotics (omeprazole, amoxycillin, clarithromycin, and metronidazole) was used to treat *H. pylori* infection. The results showed that the control group without probiotics had an 86.8 percent cure rate for *H. pylori*, while the experimental group with probiotics had a cure rate of 92 percent (Viazis et al., 2022). Aiba et al. treated a model mouse infected with *H. pylori* with *L. johnsonii* No. 1088. The study showed that *L. johnsonii* No. 1088 significantly blocked the growth of *H. pylori* in the stomachs of mice (Aiba et al., 2017). Therefore, probiotics are another useful alternative treatment for *H. pylori* infection.

Different probiotics inhibit *H. pylori* through different pathways, and the probiotics that can treat *H. pylori* are listed in Table 2. Probiotics not only inhibit the activity of urease, but also inhibit the adhesion of *H. pylori* to host cells, which is the key to probiotics in treating *H. pylori* infection (Thuy et al., 2022; Wu et al., 2023). Therefore, probiotics are potentially vital in preventing *H. pylori* infection. The results suggest that *H. pylori* infection can be treated through diet. Probiotics have been an important tool to treat *H. pylori*, and the future development should provide further opportunities for the use of probiotics.

**Table 2:** The type of probiotic that inhibits *Helicobacter pylori*.

Probiotics	Source	Function	Effect	Ref.
*Lactobacillus reuteri* DSM 17648	Probiotic capsule	Improving the eradication rate of *H. pylori* and reducing symptoms such as abdominal discomfort	The probiotic group had an eradication rate of 91.1% compared to the placebo group (68.9%)	Ismail et al. (2023)
*Lactobacillus johnsonii* No. 1088	Gastric juice	Have the strongest acid resistance; inhibit the growth of *H. pylori*	When co-cultured with *L. johnsonii* No.1088, the level of *H. pylori* decreased to about 1/3,000	Aiba et al. (2015)
*Lactobacillus salivarius* LN12	——	Destroy the biofilm of *H. pylori* and inhibit the growth of *H. pylori*	After probiotic treatment, the morphology of the *H. pylori* biofilm changes from a helical arrangement to a loose, broken and cracked one	Jin and Yang (2021)
*Lactobacillus acidophilus* NCFM	Compound *Lactobacillus* Tablets	Reduce the adhesion of *H. pylori* to AGS cells and reduce the occurrence of stomach inflammation	The mRNA and protein expression levels of pro-inflammatory cytokines (IL-8 and TNF-α, etc.) in probiotic group were significantly inhibited. The urease activity (urea A and urea B) in the treatment group decreased significantly	Shen et al. (2023)
*Lactobacillus plantarum* ZJ316	Fresh fecal samples from children	Reduce the secretion of interleukin-6 (IL-6), promote the release of IL-10, and repair mucosal damage	*L. plantarum* ZJ316 could inhibit the urease activity of *H. pylori* with an inhibitory rate of 67.47% ± 2.36%	Zhou et al. (2021)
*Lacticaseibacillus casei* T1	——	Reduce the oxidative stress caused by *H. pylori*, improve the inflammatory reaction and reduce the damage of gastric mucosa.	The expression levels of pro-inflammatory cytokines, such as IL-6 and TNF-α, were significantly decreased in the probiotic group	Yu et al. (2023)
*Lactiplantibacillus pentosus* SLC13	Mustard pickles	Modulat inflammatory response, reduce urease activity and attachment on the cells	The cell-free supernatant of *L. pentosus* SLC13 inhibited the growth of 78% of *H. pylori*	Thuy et al. (2022)
*Lactobacillus rhamnosus* JB3	Dairy product	Interfere with *H. pylori* pathogenesis	*L. rhamnosus* JB3 inhibited the expression of VacA gene in *H. pylori* cells with multiplicity of infections (MOI) of 25 and 50	Do et al. (2021)
*Lactobacillus fermentum* UCO-979C	Human gastric tissue	Inhibition of the production of proinflammatory cytokines in AGS cells has a beneficial anti-inflammatory effect	After probiotics treatment, the contents of pro-inflammatory cytokines such as TNF-α, IL-1β, IL-6, and IL-8 in AGS cells decreased significantly	Garcia-Castillo et al. (2018)

#### 3.3.1 Treatment mechanism of probiotics

The possible mechanism of probiotics to inhibit *H. pylori* infection is mainly through the following two ways.

##### 3.3.1.1 Non-immune pathways

(1) Probiotics can inhibit *H. pylori* by produce antibacterial substances. Studies have shown that probiotics can produce various antibacterial substances that affect the growth of *H. pylori*, such as hydrogen peroxide, organic acids and bactericin (Homan and Orel, 2015). The *Bulgarian* strain was found to produce an antibacterial substance with a strong anti-*H. pylori* effect activity, inhibiting more than 81% of *H. pylori* (Boyanova et al., 2017). Treatment with *L. reuteri* ATCC 23272 and its supernatant reduced *H. pylori* by 62.5 and 100%, respectively. Probiotics can also inhibit urease activity, but in a neutral environment this inhibition is removed. Thus, it is conjectured that the supernatant contains acids. To confirm this conjecture, Rezaee et al. culture *H. pylori* in the same environment using lactic acid and find that the level of inhibition of urease is similar to that of supernatant. It thus provides additional evidence that *L. reuteri* ATCC 23272 produces antibacterial acids (Rezaee et al., 2019). (2) Some probiotics prevent *H. pylori* from adhering to cells in the host’s gastrointestinal tract (Goderska et al., 2018). Probiotics can prevent *H. pylori* infection by synthesizing antimicrobial agents. In addition, probiotics can also compete with *H. pylori* at the junction with the host cell, reducing *H. pylori* adhesion. Organic acids are antibacterial substances, they can enter the body of *H. pylori* and reduce the pH, causing the death of the bacteria (Huang et al., 2022). *Saccharomyces boulardii* CNCM I-745 can block the combination of *H. pylori* with host cells (mainly duodenal cells). The reason may be that the probiotic contains an amidase that regulates the adhesion of *H. pylori* to host cells (Czerucka and Rampal, 2019). Thus, *Saccharomyces boulardii* holds great promise for the treatment of *H. pylori*. *L. plantarum* ZJ316 was effective in inhibiting the adhesion of *H. pylori* to AGS cells, reducing adhesion by 70.14% (Wu et al., 2023). When Shen et al. cultured *L. acidophilus* NCFM and *L. plantarum* Lp-115 with AGS cells (*H. pylori* positive), it was found that probiotics hinder the adhesion of *H. pylori* to host cells (Shen et al., 2023). (3) Urease is one of the indispensable factors of *H. pylori* colonization in the digestive system, which is composed of Ure subunits (A, B, C). The enzyme can break down urea into ammonia, which neutralizes the gastric environment. *L. plantarum* ZJ316 blocks the expression of the Ure gene, thereby inhibiting the synthesis of urease (Wu et al., 2023).

##### 3.3.1.2 Immune pathways

Different probiotics have different effects on the immune system. (1) Some probiotics also induce the production of anti-inflammatory cytokine (IL-10) and inhibit the secretion of pro-inflammatory cytokine (IL-6, IL-1β, INF-γ), which mediates the inflammatory response *in vivo* (Thuy et al., 2022; Forooghi Nia et al., 2023). Mice infected with *H. pylori* were fed *L. rhamnosus* LGG-18 and *L. acidophilus* Chen-08. The results showed that probiotics could significantly hinder the expression of pro-inflammatory factors related genes (NF—kappa B, TNF signaling pathway related genes; He et al., 2022). Forooghi Nia et al. treated mice positive for *H. pylori* with *Limosilactobacillus reuteri* 2892. After 5 weeks, the results showed that the secretion of cytokines such as IL-6, IL-1β, and INF-γ decreased significantly, while the secretion of IL-10 was significantly increased (Forooghi Nia et al., 2023). (2) Probiotics can enhance both the humoral and cellular immune responses in the host by modulating phagocytes and lymphocytes (Baryshnikova et al., 2023).

In addition, the combination of probiotics and herbs not only improves the fermentation effect of live bacteria, but also treats *H. pylori* infection and considerably improves the gastrointestinal health of humans. Hasna et al. treated *H. pylori* with fenugreek extract and *Bifidobacterium breve* alone, and the highest IZD (inhibition zone diameter) was 16.00 ± 0.00 mm and 20.33 ± 0.58 mm, respectively. However, when the two drugs were combined to treat *H. pylori*, the IZD was 28.67 ± 0.58 mm (Hasna et al., 2023). As a result, the combination began to be taken seriously. However, probiotic therapy requires specific clinical data to verify its cure rate and efficacy, and thus requires further validation.

### 3.4 Treatment based on nano-delivery technology

In order to deliver drugs to *H. pylori* colonization sites more effectively and improve the eradication rate of *H. pylori*, it is necessary to develop a drug delivery system to prevent the acid environment in the stomach from damaging the drug (Luo et al., 2018). Nanoparticles, usually less than 100 nm in size, are the most commonly used delivery carriers. Nanoparticles have large specific surface area and can carry additional drugs to reach the target. They are mainly bound to drugs through chemical bond interactions, adsorption, or embedding. As a result, this delivery method can protect the drug from stomach acid and reduce resistance, thus prolonging the time of action of the drug at the target and improving the therapeutic effect of the drug (Sousa et al., 2022).

**Figure 6:**
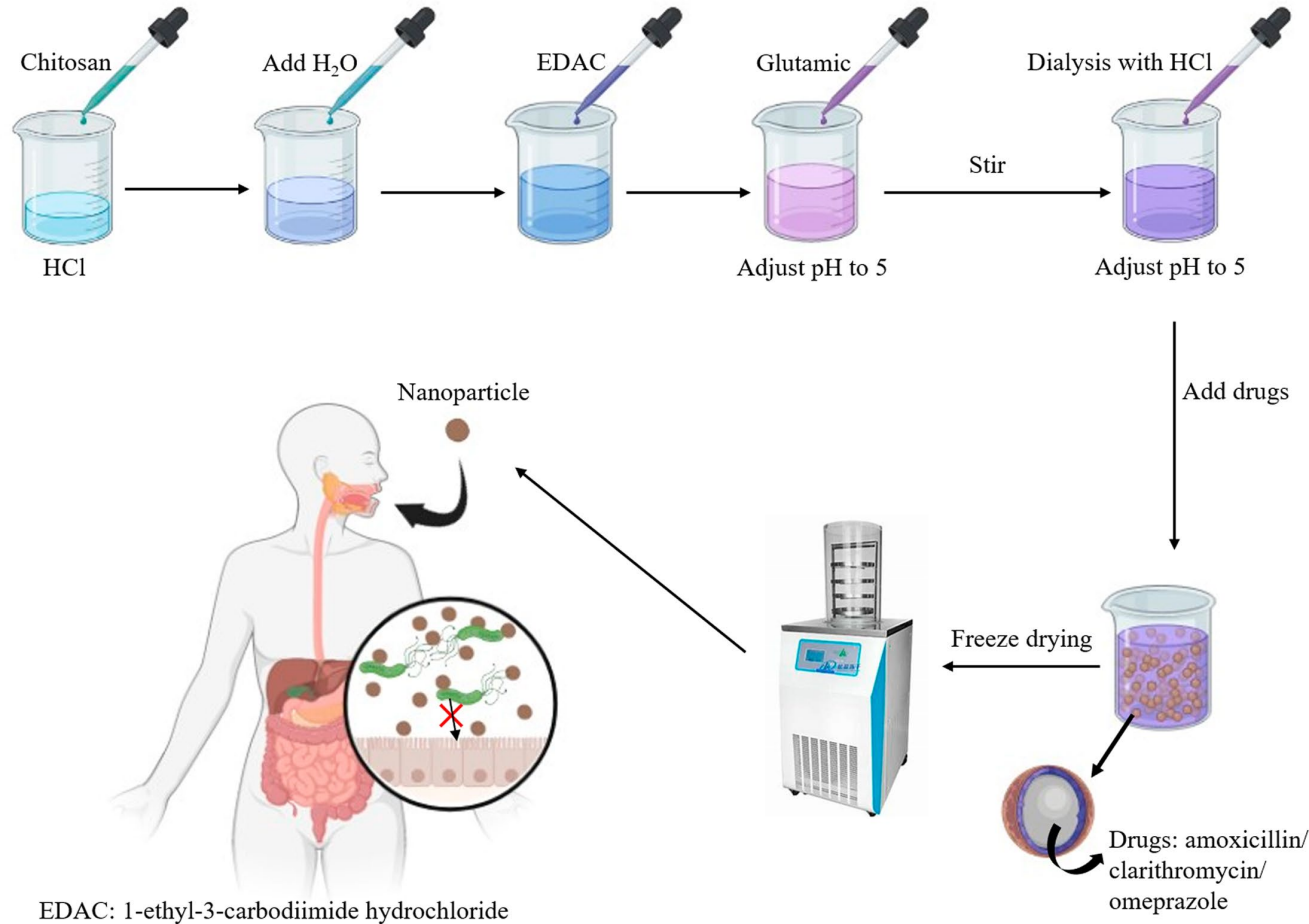
Picture of drug encapsulation.

Chitosan is the most commonly used carrier for nano-delivery systems. It can penetrate pores in the mucous layer to reach the surface of the gastric epithelium and deliver drugs to the infection site of *H. pylori* for treatment (Sun et al., 2022). Chitosan has excellent biocompatibility and adheres efficiently to the gastric mucosa system, which prolongs the administration time of the drug in the target cells (Sousa et al., 2022). The development of mucoadhesive nanoparticle delivery systems, such as the chitosan-glutamate nanoparticle system based on amoxicillin and clarithromycin, has been intensively investigated. The system encapsulates amoxicillin and clarithromycin in nanoparticles that protect the antibiotics from gastric acid destruction and adhere to the gastric mucus layer to prolong drug retention time. The production process is illustrated in Figure 6. The eradication rate of *H. pylori* in the system is 97.17%. The drug release time can be extended by up to 5–8 h (Ramteke et al., 2009). DHA is an unsaturated fatty acid that disrupts the structure of the cell membrane of *H. pylori* and has a strong bactericidal function. Chitosan is an effective carrier of nano-delivery systems that contain antibiotics to resist damage from stomach acid. Khoshnood et al. therefore designed a nanoparticle based on chitosan and alginate’s DHA (docosahexaenoic acid) -AMX (amoxicillin) for the treatment of *H. pylori*. Nanoparticles supplemented with 2% (v/v) DHA significantly hindered the growth of *H. pylori* compared to the control group (no nanoparticles added). Moreover, the inclusion rate of antibiotics increased to 76 percent after the addition of this fatty acid (Khoshnood et al., 2023). *In vivo*, fucus-chitosan/heparin nanoparticle delivery systems significantly increased the ability to inhibit *H. pylori* compared to conventional antibiotic therapy. The delivery system (containing 6.0 mg/L of berberine) had an inhibition rate approximately 25.9% ± 3.7% higher than that of the control group (Lin et al., 2015). In addition, the delivery system can encapsulate other non-antibiotic substances, such as antimicrobial peptides and phenolic compounds, to reduce the emergence of resistant strains (Zhang et al., 2015). Nanoparticle-based drug delivery systems have a promising future as they can effectively treat *H. pylori* infection and are commonly used in the food industry to develop foods to treat *H. pylori* (Sun et al., 2022).

Liposomes are another drug delivery system that has been successfully used. They have many advantages, such as high encapsulation rates, high safety and biocompatibility (Sharaf et al., 2021). When *H. pylori* forms a biofilm in the mucous membrane of the stomach, it develops antibiotic resistance, allowing bacteria in the mucous membrane to continue to infect (Shen et al., 2020). Berberine, a substance isolated from *Coptis chinensis*, has the activity of inhibiting *H. pylori* urease. Previous studies have confirmed that berberine in combination with antibiotics can be used in the treatment of *H. pylori*. In addition, the alkaloids destroy bacterial biofilms, which reduces microbial resistance. The positively charged berberine derivative (BDs) synthesized by Shen et al. did not easily pass through the negatively charged mucosa, so it was difficult to enter the *H. pylori* aggregation site. Shen et al. designed a BDs-Rhamnose-lipids (RHL) based nano-drug delivery system that can penetrate the mucous layer to reach bacterial aggregation points. The clustering rate of the BDs-RHL delivery system is roughly three times higher than that of the BDs delivery system. The MIC value of the system containing BDs (decocarb) was 1.56 μg/mL, while that of the BDS-RHL system was 0.78 μg/mL. The effectiveness of drug treatment also improved considerably as more of the drug reached the site of bacterial infection (Shen et al., 2020). Many nanoscale lipid carriers have been developed to deliver drugs in radical *H. pylori* therapy. Using liposomes to deliver hesperidin and clarithromycin has been shown to treat *H. pylori* infection with inhibition rates of up to 94% (Sharaf et al., 2021). A lipid carrier based on mannosylerythritol lipid-B can carry amoxicillin through the mucous layer of the stomach of mice (infected with *H. pylori*), eliminating the inflammation of the mucous layer (Wu et al., 2022). As shown in Table 3, these delivery systems have a suitable size range and can penetrate the viscous layer. They are also much more adhesive, thus increasing the retention time of the drug, which is effective in treating *H. pylori* infection. These systems, which maximize the release of drugs outside the body, promise to replace conventional therapies. As a result, nano-delivery systems can be used to treat *H. pylori* infection and have great promise in food, medicine and other fields.

**Table 3:** Several drug delivery systems have been developed.

Delivery system	Preparation method	Encapsulated compound	Size	effect	Advantages (+) or disadvantages (−)	Ref.
Chitosan nanoparticles based on sodium alginate-polyethylene glycol	Ionotropic gelation	Ovalbumin as model antigen	211 ± 5–319 ± 5	The highest adhesion rate *in vitro* was 63%	+ Sodium alginate embedded nanoparticles have higher mucin binding level	Amin and Boateng (2022)
Sterculia foetida and pullulan-based semi-interpenetrating polymer network gastroretentive microsphere (Chitosan system)	Emulsion crosslinking	Amoxicillin,Trihydrate	57.99 ± 1.53–121.90 ± 1.38 μm	Drug entrapment efficiency: 88.75 ± 1.18%Mucoadhesion rate: 81.73 ± 1.50%,Drug release rate: 80.43 ± 1.2%	+ Acid resistant, long retention time in the stomach,– The encapsulation efficiency is affected by glutaraldehyde concentration	Hadke and Khan (2021)
Carbopol-loaded amoxicillin nanospheres (Chitosan system)	Spray drying	Amoxicillin	280–320 nm	Yield: 92.8% ± 0.9%	+ High stability in 15°C–25°C,– Clumping is observed at 37°C	Harsha (2012)
Mannosylerythritol Lipid-B-Phospholipidnanoliposome (Lipid system)	Thin-film hydration methods with ultrasonication	Amoxicillin	100 nm	Drug entrapment efficiency: 65%,Drug release rate: 84.6%	+ Acid resistance	Wu et al. (2022)
Nanostructured lipid carrier (Lipid system)	Thermal homogenization and ultrasonic methods	Hesperidin (Hesp), clarithromycin (CLR)	221–638 nm	Drug entrapment efficiency _CLR and Hesp_: 13%–28%,Inhibition rate: 94.34% ± 3.68%	+ Biocompatibility, stable in water	Sharaf et al. (2021)

### 3.5 Other emerging therapies

In addition to the mentioned plant and microbial therapies, there are a number of different treatments on the rise.

#### 3.5.1 Lactoferrin

Lactoferrin can bind to iron ions, inhibiting the survival of microorganisms in the absence of iron. After *H. pylori* entered human body, lactoferrin content in stomach increased significantly (Imoto et al., 2023). Lu et al. used animal models to investigate whether infection with *H. pylori* affects the lactoferrin content in the host. The results showed that the expression level of lactoferrin in the stomach infected with *H. pylori* was 9.3 times that of the uninfected group (Lu et al., 2021). *H. pylori* contains a T4SS system that delivers virulence factor (CagA) to target cells and causes inflammation in the host. Iron ions have antibacterial effects by affecting the activity of the T4SS system (Lu et al., 2021). Therefore, lactoferrin is an excellent antimicrobial. Bovine lactoferrin (concentration between 25.2–50.0 mg/mL) can completely inhibit the growth of *H. pylori in vitro* (Imoto et al., 2023). Previous studies have confirmed that lactoferrin alone does not eliminate bacterial colonization when used in the treatment of *H. pylori* infection (Imoto et al., 2023). For this reason, lactoferrin is commonly used in combination with different drugs to treat *H. pylori* infection. Bovine lactoferrin has an inhibitory effect on all *H. pylori in vivo* with a MIC of 5–20 mg/mL. When the protein was combined with antibiotics (levofloxacin) to treat *H. pylori* infection, the MIC value (0.31–2.5 mg/mL) decreased significantly (Ciccaglione et al., 2019). *In vitro*, patients infected with *H. pylori* were treated with antibiotics (esomeprazole, amoxicillin, levofloxacin) and bovine lactoferrin. The eradication rate of *H. pylori* in the antibiotic group alone was 75%, while that in the antibiotic combined with bovine lactoferrin group was 92.8% (Ciccaglione et al., 2019). Hablass et al. designed antibiotic therapy (clarithromycin + amoxicillin/metronidazole +PPI) in combination with bovine lactoferrin to treat *H. pylori* positive volunteers. The combined treatment group had a successful eradication rate of 85.6%, compared with 70.3% for the lactoferrin-free group, suggesting that lactoferrin may improve the efficacy of antibiotic therapy (Hablass et al., 2021). In summary, the use of lactoferrin in the treatment of *H. pylori* infection has been shown to be effective in increasing the success rate. The combination of lactoferrin and antibiotics may be a useful alternative to triple therapy in the future.

#### 3.5.2 Phages

Phages invade bacteria, using the nucleic acid of the host cell to replicate and produce fresh phages that lyse the host cell, thus acting as antimicrobials (Sousa et al., 2022). Yahara et al. showed that about one-fifth of *H. pylori* contains prophage genes (Yahara et al., 2019). Using phages to treat related diseases is safer and more reliable than conventional antibiotic therapy. Numerous phages target bacteria and can specifically destroy strains. Phages do not invade animal cells or even humans, so phage therapy is considered safe (Sousa et al., 2022). Cuomo et al. demonstrated the activity of *Hp* (*H. pylori*) phage in inhibiting the growth of strains of *H. pylori*. In addition, they used antibacterial lactoferrin to design a nano-system based on *HP* phage-lactoferrin hydroxyapatite. The bacteriostatic effect of the system is better than that of the phage group alone (Cuomo et al., 2020). Therefore, phages have been shown to be beneficial in the treatment of *H. pylori* contamination, especially in combination with other antimicrobial agents. However, there are few studies of phage therapy, so further data and studies are needed to support this approach as a first-line treatment.

#### 3.5.3 Vaccine

Vaccination is a powerful tool for treating patients infected with *H. pylori.* Univalent vaccine is not as useful as multivalent vaccine in the treatment of *helicobacter pylori* infection (Guo et al., 2017). Studies have designed a multivalent epitope vaccine using urease polypeptides with immune adjuvants, CagA and VacA, to evaluate the efficacy of the vaccine against *H. pylori* infection in mice. The results showed that the vaccine promoted the production of more specific antibodies to CagA, VacA and immune adjuvants. In addition, adding polysaccharide adjuvant to the polyvalent vaccine group significantly reduced *H. pylori* levels in the stomachs of mice compared to the monovalent vaccine group (Guo et al., 2019). As a result, multivalent vaccines are becoming increasingly popular in medicine. Guo et al. designed a multivalent epitope vaccine using *H. pylori* adhesion molecules (urease, Lpp20, HpaA and CagL) and investigated the therapeutic potential of the vaccine in animal models infected with *H. pylori*. The results showed that the vaccine produced additional antibodies against the adhesion molecules in the mice (Guo et al., 2017). It has been reported that inactivated *H. pylori* whole-cell vaccine can reduce the colonization of *H. pylori* in the stomach by enhancing human mucosal immunity (Zhang et al., 2022). There is also a vector vaccine that has achieved excellent results in oral immunization. Katsande et al. used spores derived from *Bacillus subtilis* to design a vector vaccine expressing urease subunits (A and B), which was used orally to treat an animal model of *H. pylori* infection. The final results showed an increase in IgA levels and a significant reduction in the amount of *H. pylori* colonizing the mice’s stomachs after oral treatment (Katsande et al., 2023). In addition, a nano-delivery system based on N-2-hydroxypropyl trimethyl ammonium chloride chitosan/carboxymethyl chitosan was designed and used as an immune adjuvant to treat *H. pylori* infection in animal models. The results showed that the expression of pro-inflammatory cytokines (IL-6, IL-4, etc.) in mice increased significantly after the nano-drug treatment, which indicated that the nano-system could effectively promote the occurrence of immune response (Gao et al., 2022).

#### 3.5.4 Phototherapy

Phototherapy is the use of a laser to treat a laser-sensitive substance that secretes the bacteriocidal agent ROS after being stimulated. Also, the therapy does not create resistant strains as easily as antibiotics do. And ROS produced after laser irradiation can destroy the cell membranes of bacteria, causing the pathogenic bacteria to crack and die. Therefore, this therapy is a promising treatment for *H. pylori* (Im et al., 2021). Some studies have designed a photosensitive substance based on 3′-sialyl lactose coupled poly (L-lysine), which was orally infected with *H. pylori* in mice. The mice were treated with a gastroscope laser system. The content of *H. pylori* in the stomach of mice was significantly reduced when the laser treatment was over 1.2 J cm^−2^, and it was fully inhibited when it was over 2.4 J cm^−2^. In addition, when mice were treated with 4 J cm^−2^ laser, no damage to AGS cells was observed (Im et al., 2021). Phototherapy can destroy the biofilm of *H. pylori*. As such, it has a positive therapeutic effect on antibiotic-resistant strains. Because the surface of biofilm contains anions, Qiao et al. developed a new microbial targeted near-infrared photosensitive substance based on guanidine (positively charged) and photosensitizer to inhibit the growth of *H. pylori*. After laser treatment, the bacterial biofilm density was significantly less than before laser treatment, suggesting that phototherapy can considerably damage bacterial biofilms (Qiao et al., 2023). *H. pylori* was treated *in vitro* with a blue light-emitting diode. The results showed that after 6 min of treatment, the activity of urease produced by *H. pylori* was inhibited. In addition, more than half of the *H. pylori* biofilm was damaged after phototherapy compared to the group without phototherapy (Darmani et al., 2019). Curcumin and blue light emitting diodes have been used to treat *H. pylori*. The results showed that the number of *H. pylori* was significantly suppressed in the curcumin + blue light treatment group compared to the group without blue light irradiation (Darmani et al., 2020). Xiao et al. designed an antibody nanoprobe (gold nanostar coupled acid-sensitive cis-aconite) for *in vivo* infection with *H. pylori*. The probe killed all *H. pylori* bacteria in mice treated with near-infrared light. All of the probes were regularly excreted 7 days after entering the mice. Gastrointestinal symptoms caused by *H. pylori* gradually disappear within a month (Zhi et al., 2019). This approach remains promising for the successful treatment of *H. pylori* infection.

## 4 Conclusions and future prospects

*Helicobacter pylori* infection is currently a non-negligible problem. The bacterium can colonize the human gastrointestinal tract and significantly increase the risk of stomach cancer in humans. Previous antibiotic treatments have caused resistance to *H. pylori* strains around the world, so an alternative treatment is being sought. Therefore, this paper focuses on the prevention and treatment of *H. pylori*, encompassing an in-depth exploration of its pathogenic mechanisms, transmission routes, and emerging therapeutic interventions. Based on *H. pylori* studies, we summarize the current status and treatment mechanisms of seven approaches, including phytotherapy, probiotic therapy, nano-delivery therapy, lactoferrin therapy, phage therapy, vaccine, and light therapy. The safety and non-resistance properties of phytotherapy and probiotics have been demonstrated by numerous studies, rendering them the preferred choice for second-line treatment. The use of nano-systems to deliver drugs can address the short retention time of drugs in the stomach, which considerably improves drug utilization. Lactoferrin therapy itself is safe and pollution-free, and is an excellent alternative therapy. Current phage therapies and vaccines exhibit targeted efficacy, yet their clinical application necessitates further investigation due to the limited availability of clinical data. Light therapy is still in its infancy, with limited research data and certain safety risks. All emerging therapies have achieved excellent results, but additional investigations are needed due to the lack of studies on the treatment mechanisms and clinical data.

## Author contributions

ML: Data curation, Investigation, Software, Writing – original draft. HG: Formal Analysis, Software, Writing – review & editing. JM: Supervision, Writing – review & editing. ZiZ: Writing – review & editing. LZ: Writing – review & editing. FL: Writing – review & editing. SZ: Writing – review & editing. ZhZ: Writing – review & editing. SL: Writing – review & editing. HL: Writing – review & editing. JS: Funding acquisition, Supervision, Writing – review & editing.
